# A Comparison of Four Newer Supraglottic Airway Devices for Airway Management of Entrapped Trauma Patients with Difficult Access—A Randomised, Controlled Manikin Trial

**DOI:** 10.3390/life15121904

**Published:** 2025-12-12

**Authors:** Dawid Aleksandrowicz, Tomasz Gaszyński

**Affiliations:** 1 Department of Anaesthetics, Intensive Care Medicine and Pain Therapy, Mazovian Specialist Hospital, University of Radom, 26-600 Radom, Poland; 2 Department of Anesthesiology and Intensive Therapy, University of Łódź, 90-153 Łódź, Poland; tomasz.gaszynski@umed.lodz.pl

**Keywords:** supraglottic airways, entrapped patients, road traffic accidents, restricted access, cervical spine immobilisation

## Abstract

Background: Airway management forms the most important component of pre-hospital trauma patients’ care. In such a setting, the definitive airways may be difficult to establish. This study aimed to evaluate four newer supraglottic airway devices in a simulated condition of an entrapped trauma patient with restricted access. Methods: An intubation manikin with a cervical collar on was placed on the driver’s seat of a passenger car, which was positioned on its left side. The access to the manikin was only allowed from the front. The insertion to successful ventilation (T_iv_) time was recorded. First-pass success and the ease of use were also evaluated. Results: The AuraGain device required the shortest median T_iv_ at 7.5 s (Interquartile Range, IQR 4) vs. 9.5 s (IQR 11), *p* < 0.001. The AuraGain achieved the highest first-pass success—90%. The Baska mask was the most user-friendly, achieving a mean score of 8.6. Conclusions: The AuraGain outperformed other studied devices concerning the T_iv_ as well as the first-pass success. The Baska mask was the easiest to use.

## 1. Introduction

Airway management forms the most important component of pre-hospital trauma patients’ care, both in the adult as well as paediatric populations [1]. Failure in the maintenance of airway patency will lead to rapid deterioration and ultimately the patient’s death. On the other hand, pre-hospital airway management may be challenging to perform and associated with serious complications [2]. This is in particular true in certain patient populations, such as entrapped, i.e., RTA (road traffic accident), victims who are unable to exit the vehicle unassisted [3].

Historically, definitive airways, with tracheal intubation in particular, have been considered the gold standard in regard to airway management. Various definitions of definitive airways exist in the literature [4,5]. Common elements include a cuffed tracheal tube placed in the trachea with the cuff inflated below the vocal cords. They offer the highest level of protection from aspiration, enable adequate ventilation, and allow for the effective removal of secretions. However, in the pre-hospital setting, success rates of tracheal intubation vary, and high rates of complications have been described in the literature [6,7]. Furthermore, definitive airways require a higher level of manual skills, good hand–eye coordination, and distinct expertise. The presence of saliva, blood, or vomit may worsen intubation conditions in already challenging and difficult settings. SADs have a steep learning curve, allowing for fast skill acquisition. This, in turn, makes them a useful tool in the hands of skilful paramedics.

It is worth noting that definitive airways might be difficult or even impossible to establish in trauma patients, especially in challenging pre-hospital settings [8]. Therefore, supraglottic airway devices (SADs) may be an alternative in such patients. SADs have been successfully used both in-hospital as well as in out-of-hospital settings since the mid-1980s. Since their introduction into clinical practice, they have become a crucial and important component of various airway management approaches worldwide. Furthermore, an SAD is not only an airway device per se but may also be used as a conduit for intubation [9].

The first SAD used in modern-day clinical practice was the Laryngeal Mask Airway (LMA) developed in the first half of the 1980s [10]. Since then, the modifications of the original single-lumen design of an SAD have led to various new types and generations of supraglottic airway devices, such as double-lumen, e.g., the I-gel, or intubating SADs, e.g., the iLTS-D (Intubating Laryngeal Tube Suction—Disposable). There is no single ideal type of supraglottic airway device that can be used universally in every clinical scenario. This means that new designs and types of SADs are still being developed and appearing on the market. This, in turn, necessitates their evaluation in different scenarios, both in hospital and out of hospital. Such an approach is necessary to identify the SADs that perform best in different clinical situations.

One of the most challenging situations regarding airway management in the pre-hospital setting is a trauma patient with restricted access. Definitive airways may be very difficult to establish in such patients. A good alternative to tracheal intubation could be SADs [11,12]. While some of the older devices, such as the classic LMA, the LMA Supreme, and the I-gel, have been thoroughly investigated in different settings, the data in the current literature on more recent or newer supraglottic airway devices in entrapped trauma patients are rather scant [13].

According to the recent World Health Organization (WHO) report, the number of road traffic accidents is on the rise [14]. As a result of this, it will become more likely to deal with a trauma patient who is entrapped in the vehicle. It is of the utmost importance to provide appropriate pre-hospital care to those who are not able to exit the vehicle unassisted. Airway management forms one of the most important elements of patients’ care in such circumstances, and SADs may be the first-choice tool in such situations.

The aim of this study was to evaluate four SADs, the Baska mask (Baska Versatile Laryngeal Mask Pty Ltd., Strathfield, Australia), the iLTS-D (VBM Medizintechnik GmbH, Sulz am Neckar, Germany), the AuraGain (Ambu A/S, Ballerup, Denmark), and the DM Safety Double-Lumen Laryngeal Mask (Dami Medical Ltd., Hefei, China), for ventilation in a simulated condition of an entrapped trauma patient with simultaneous cervical spine immobilisation. All airway devices were evaluated by experienced paramedics in a situation where there was difficult access to the RTA victim.

## 2. Materials and Methods

This study has been approved by the University of Radom Ethics Committee (KB/08/2025, head: Prof. J. Jońska-Gmyrek, date: 12 February 2025). Trial registration Deutsches Register Klinischer Studien: DRKS00038249 (registration date: 4 November 2025). Our research was initially registered at the Clinical Trials. However, due to the Government Shutdown in the USA, we were unable to make any further updates regarding your study as the registry was inactive during the period of the shutdown. In view of the complex situation within the Clinical Trials registry, we decided to re-register the study at the Deutsches Register Klinischer Studien which was done in October but due to their formal proceeding was only officially confirmed on 4th November.

**Figure 1:**
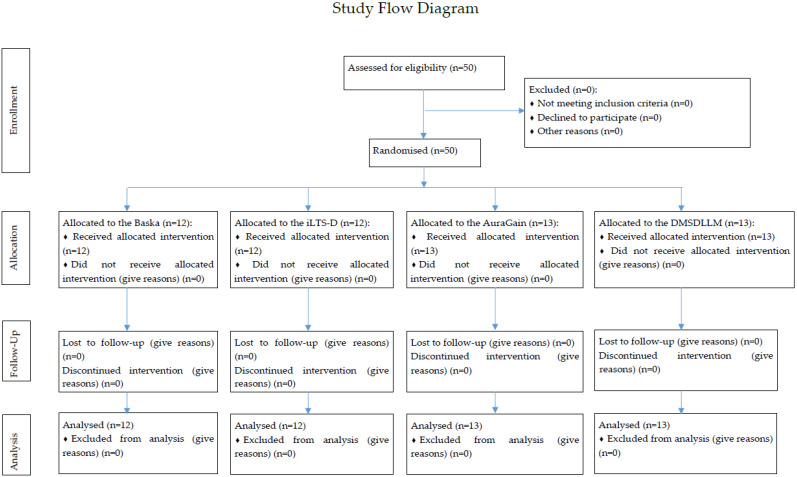
Study flow diagram.

Written informed consent was obtained from all study participants. Fully qualified paramedics were enrolled in this study. Their experience varied between six and eight years of practice after completion of paramedic training. All study participants had never used the SADs under study. The study flow diagram is shown in Figure 1.

**Figure 2:**
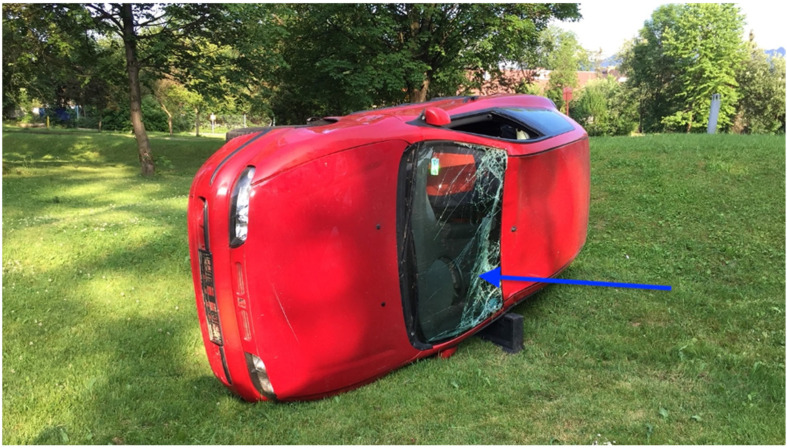
The final position of the passenger car used for this study (please note that the windscreen was removed prior to the evaluation of the SADs). The blue arrows indicate the manikin’s position.

**Figure 3:**
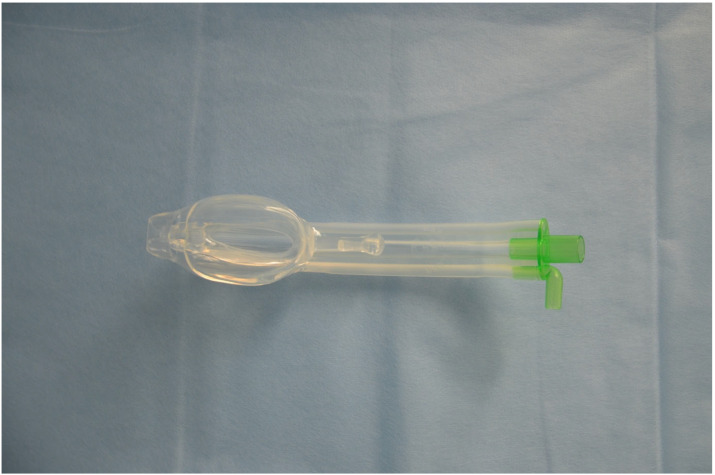
The Baska mask.

**Figure 4:**
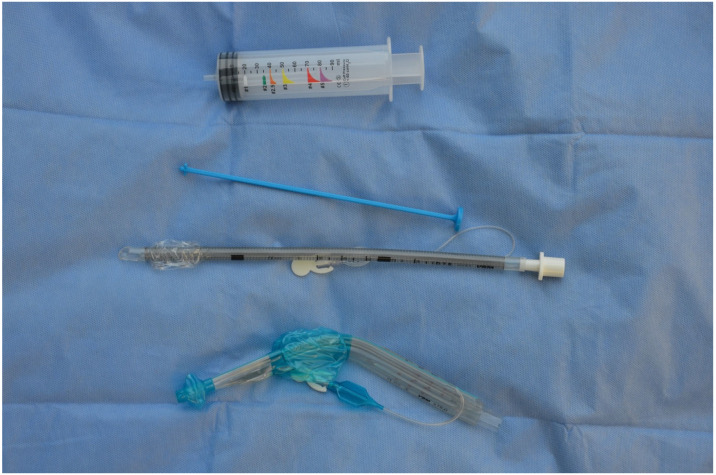
The iLTS-D.

**Figure 5:**
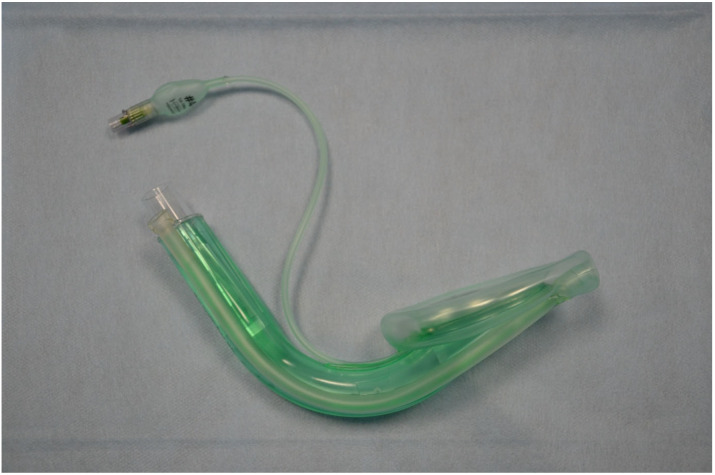
The AuraGain.

**Figure 6:**
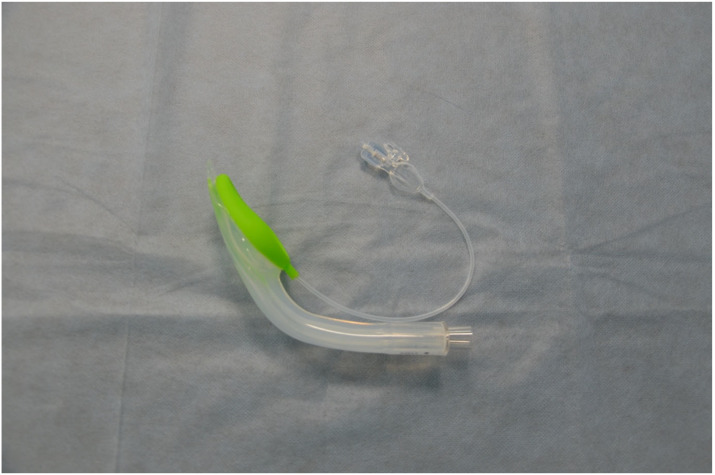
The DM Safety Double-Lumen Laryngeal Mask.

A 10 min long lecture was delivered before the start of this study. Its aim was to explain how to use the evaluated devices. A 30 min practice session was allowed following the lecture, during which all study participants could familiarise themselves with the equipment and practice with it. A skill station was set up containing an AT Kelly Torso intubation manikin (Laerdal Medical AS, Stavanger, Norway). A Patriot^®^ cervical collar (Össur hf., Reykjavik, Iceland) was applied in order to achieve cervical spine immobilisation. After completion of the initial practice, the manikin with the cervical collar on was placed on and secured to the driver’s seat of a mid-sized passenger car (FIAT S.p.A., Turin, Italy). Firemen from a local fire brigade positioned the car on its side and secured it in place (Figure 2). The windscreen was removed, and an opening was created through which access to the manikin was allowed. A unique number was allocated to each of the four evaluated SADs: 1 for the Baska mask (Figure 3), 2 for the iLTS-D (Figure 4), 3 for the AuraGain (Figure 5), and 4 for the DM Safety Double-Lumen Laryngeal Mask (Figure 6). A single sheet of paper containing a digit/number printed on it was placed in a dark, opaque envelope. The SPSS 29.0 software (IBM Corp., Armonk, NY, USA) was used for random allocation of these sheets. Each study participant randomly picked up an envelope and then was provided with the corresponding supraglottic airway device to use. The maximum number of insertion attempts was three per device. The primary outcome was the time required to insert the device and achieve successful ventilation (T_iv_). This was measured using a stopwatch on a mobile phone (Apple Inc., Cupertino, CA, USA). The secondary outcomes were the efficacy of the studied devices and the ease of use. The latter was measured using an 11-point numerical rating scale (NRS), where 0 corresponded to a very difficult to use device and 10 indicated an easy to use and user-friendly device. A manual resuscitator (Ambu A/S, Ballerup, Denmark) was used for ventilation and was readily available to the paramedics. The study participants performed all insertions with each of the four SADs. Failed insertion and ventilation was defined as an insertion attempt that lasted longer than two min (120 s) or an attempt that resulted in failed manikin ventilation. Only those participants who failed to insert/ventilate were allowed another attempt. Microsoft Office Excel 2021 spreadsheet (Microsoft Corporation, Redmond, WA, USA) and Statistica 14.0 (TIBCO Software, Inc., Palo Alto, CA, USA) were used to analyse the collected data. The Kolmogorov–Smirnov test was utilised to determine whether the analysed variables matched the characteristics of a normal distribution. A paired Student *t*-test and the Wilcoxon signed-rank test were used for data analysis. Based on our previous study, we assumed that the overall success rate of intubation in trauma patients with a difficult airway would be 90% (α = 0.05, 2-sided, β = 0.1, 95% CI, *t*-value ± 2.31), and the calculated sample size required 45 participants. The final adjusted sample size was 50 participants, allowing a drop-out rate of about 10%. A *p*-value of less than 0.05 (*p* < 0.05) was considered statistically significant. Cohen’s d was used for effect size calculations.

## 3. Results

Study participants consisted of fifty fully qualified, active, and experienced paramedics. Their experience ranged between six and eight years of active pre-hospital work after finishing emergency medicine training (mean 6.8 years). The majority of participants were male (*n* = 31) compared to female (*n* = 19).

The AuraGain required the shortest median T_iv_ at 7.5 s (IQR 4) vs. 9.5 s (IQR 11) for the iLTS-D, *p* < 0.001 (Table 1). The longest insertion to ventilation median time was achieved when the iLTS-D was used.

The Baska mask and the iLTS-D were the two SADs under study that required the maximum number of attempts, i.e., three, in order to achieve a 100% insertion success rate (Table 2). The use of the AuraGain was associated with the highest first-pass success rate—90%. The DMSDLLM device achieved a similar result (82%).

The iLTS-D and the AuraGain outperformed other SADs in regard to ease of use and user-friendliness. Their mean scores were 8.6 and 8.4 out of 10, respectively, both with a *p* < 0.05 (Table 3). The remaining SADs achieved significantly lower NRS scores, with the Baska mask found to be the least easy to use.

## 4. Discussion

Despite being one of the crucial elements of the pathway of care of trauma patients, airway management in the out-of-hospital setting is often difficult to perform. This difficulty is frequently further aggravated by various mechanisms of road traffic accidents, some of which may result in patients being entrapped in their vehicles. This, in turn, results in restricted access to RTA victims, making airway management and the whole resuscitation process even more difficult [15,16].

Definitive airways, such as tracheal intubation, are still considered the gold standard in regard to trauma patients [17]. However, it is worth noting that tracheal intubation is a time-consuming and high-risk procedure [7]. Furthermore, intubation may not be possible to perform in certain situations in the out-of-hospital setting. In such instances, supraglottic airway devices may be considered as an alternative, both as a temporary or permanent measure [18]. SADs have become extremely popular since their introduction to clinical practice in the late 1980s [19,20]. They have been used successfully in various clinical scenarios and have certain advantages over tracheal intubation [18,21]. Supraglottic airway devices are a vital element of difficult airway management guidelines worldwide [22,23,24]. All the above-mentioned features make SADs a useful tool in airway management, not only in hospital but also in the more challenging out-of-hospital setting.

The design of the original LMA has evolved over the years, with new double-lumen SADs being widely available and used worldwide. Other different designs have also been developed, leading to the creation of newer SADs, such as the Baska mask or the DM Safety Double-Lumen Laryngeal Mask (DMSDLLM). A large number of them have never been evaluated in the pre-hospital setting and within the trauma patient population in particular. It is of the utmost importance for an out-of-hospital care provider to be aware of which of the SADs perform best and which equipment to use in this challenging scenario. Therefore, a thorough evaluation of new devices is required.

The double-lumen SADs have certain advantages over older devices. They include the following: the presence of a gastric channel enables the decompression of the stomach (liquids and gases), thus reducing the risk of aspiration; an integrated bite-block improves the patency of the airway and minimises the risk of airway occlusion; and, finally, the improved cuff design reduces the risk of aspiration as well as enables ventilation at higher pressures [13,25]. The fact that there are numerous complications related to SADs, such as the aspiration of gastric contents, airway trauma, nerve injury, failed insertion, and the displacement of an SAD, is of note. These complications depend on age and the patient’s BMI (Body Mass Index) [26,27].

Supraglottic airway devices form a rather heterogeneous group of airway devices. There are distinct differences in the design of SADs, which include the presence of an additional suction channel, the possibility of tracheal intubation, different cuff designs, and reusability. A summary of different features of the studied devices is shown in Table 4.

The Baska mask is a relatively new SAD that is similar in its design to the I-gel. It incorporates a bite-block and a gastric channel and has an advanced self-sealing variable-pressure cuff. An insertion tap is attached to the base of the cuff and can be used to increase the curvature of the device and hence aid the insertion of the Baska mask. It comes in four sizes: 2.5, 3, 4, and 5, with the 2.5 size dedicated for ‘large children’. The appropriate age and weight are not specified, making the Baska mask rather difficult to use in the paediatric population.

The iLTS-D is a derivative of the LTS supraglottic airway device. It is designed not only to be an SAD per se but also to aid intubation. It is a double-lumen device with a dedicated gastric channel. Its unique design incorporates two separate cuffs. The iLTS-D comes with a stabiliser, which is used during intubation through the device. There are only two sizes available: 2.5/3 and 4/5. The former is dedicated to those with a height of over 125 cm, which includes older children.

The AuraGain has a significant curvature and thus is similar to the LMA Supreme. It is an example of a double-lumen SAD with an integrated gastric channel and a bite-block. There is a modified cuff design that prevents tip backfolding during insertion. The AuraGain may also be used as a conduit for intubation. It comes in the sizes 1, 1.5, 2, 2.5, 3, 4, 5, and 6, making it an SAD suitable for a wide range of patients from neonates to adults.

The DMSDLLM is a new type of SAD that resembles the I-gel. However, instead of a non-inflatable cuff, there is a more conventional solution, i.e., a pneumatic cuff. The DMSDLLM has a gastric channel as well as a novel back groove channel on the back of the device. It can also be used as a conduit for intubation. The DMSDLLM is available in the following sizes: 1, 1.5, 2, 2.5, 3, 4, and 5. It can be used for both paediatric as well as adult patients.

The aim of this study was to evaluate four newer supraglottic airway devices—the Baska mask, the AuraGain, the iLTS-D, and the DMSDLLM—for airway management with simultaneous cervical spine immobilisation in a simulated condition of an entrapped trauma patient with difficult access. All SADs under study were evaluated by fully qualified and experienced paramedics. There is a noticeable increase in the number of cars worldwide. This trend may lead to an increased occurrence of road traffic accidents with a higher probability of RTA victims being entrapped in their vehicles.

The current literature lacks a good performance of SADs within the population of the injured and regarding entrapped RTA victims in particular.

The primary outcome of this study was the insertion and ventilation time (T_iv_). The AuraGain required the shortest median T_iv_ of 7.5 s (IQR 4). The insertion and ventilation time is particularly important for airway management in those injured and with difficult access and should be performed in a rapid and efficient manner. In their study, Pap et al. evaluated four airway management devices used by paramedics in a simulated scenario of an entrapped patient [28]. Of the four assessed devices, only two were SADs, i.e., the LMA Supreme and the laryngeal tube (LT). In their study, the LMA Supreme was found to be superior in regard to the mean time to first successful ventilation. The design of the LMA Supreme is similar to the AuraGain used in our study. A distinct curvature that is anatomically shaped is present in both the AuraGain as well as the LMA Supreme. This may facilitate the insertion of both of these devices and reduce insertion times. On the other hand, such a design may impede intubation through these SADs. We found that the use of the AuraGain was associated with the fastest T_iv_. This was not only clinically but also statistically significant. Furthermore, all other SADs under study required longer insertion and ventilation times. Pap et al. achieved a longer T_iv_ with the LMA Supreme than analogous times with the AuraGain in our study, 16.7 s vs. 11.2 s. This result was similar to that of our previous study [11]. The data on the AuraGain in trauma patients are sparse in the current literature, with one case report available [29].

Intubation in out-of-hospital settings is challenging. There is a lack of studies that evaluate intubation times in road traffic accident victims or in those who are entrapped in a vehicle. In the available literature, times of pre-hospital intubation by paramedics were recorded by Shou et al. [30]. The average overall intubation time was 50 s, which is almost twice as long as the longest T_iv_ recorded in our study. The insertion of a supraglottic airway device is faster and easier when compared to tracheal intubation, and this technique should always be considered in a challenging scenario of an entrapped patient.

The first-pass success was also assessed in this study. The insertion of the AuraGain had a 90% first-attempt success. Such a good performance of this device can be partially attributed to its design, which may facilitate insertion. However, we noted a significant discrepancy regarding the first-attempt success rate during the AuraGain insertion in our previous study [11]. This may be due to a different group of paramedics who evaluated the SAD. The fact that the AuraGain outperformed all other supraglottic airway devices under study is also of note. It is also worth noting that it required a maximum of two attempts (out of the three allowed) in order to achieve an overall success rate of 100%. The iLTS-D device was the least favourable of the evaluated SADs. This device was found to require significantly more T_iv_ as well as had the lowest first-attempt success rate (78%). The iLTS-D has a unique design that not only allows ventilation and the suction of gastric contents but also facilitates intubation. However, contrary to the above-mentioned results, the iLTS-D may be a useful tool for airway management in specific trauma scenarios, as it may facilitate intubation [9,31]. Martin et al. assessed its predecessor—the King LT-D. They compared it to other intubation techniques [32]. The King LT-D outperformed other devices and techniques and offered faster insertion times and significantly higher first-pass success rates.

In our study, user-friendliness was also assessed. The standard eleven-point NRS was used to rate all of the studied SADs. A score of ‘0’ denoted the most difficult to use device. Both the Baska mask as well as the AuraGain were found to be the most user-friendly, achieving a mean NRS score of 8.6 and 8.4 out of 10, respectively. However, it has to be emphasised that an easy-to-use device does not always perform better regarding insertion and ventilation times or efficacy.

The AuraGain device outperformed other SADs in all but one study endpoint. Its performance could be related to the design of this supraglottic airway device, as there is a distinct curvature that is anatomically shaped and hence may improve its insertion. The highest success rate is usually achieved by the easiest to use device. In our study, the Baska mask narrowly outperformed the AuraGain (8.6 vs. 8.4). However, this result was neither statistically nor clinically significant.

It is rather difficult to compare the results of our study, as there is a limited number of studies available on SAD use for airway management in trauma patients, including those considered entrapped.

A large number of available studies assessed both supraglottic airway devices as well as other airway devices, such as standard or video laryngoscopes [33]. In a study by Wetsch et al., three SADs were assessed by experienced pre-hospital anaesthetists in a simulated scenario that involved an entrapped patient [34]. In their study, intubation with the standard laryngoscope was used as a reference. The laryngeal tube achieved the slowest effective ventilation time of 17.6 s (5.3). This finding was comparable to the T_iv_ achieved by the iLTS-D in our study. This could be attributed to an apparently different design of both the LT and the iLTS-D compared to other SADs used in the study. Despite these results, it has to be emphasised that the study setting was different, as access to the manikin was allowed through the driver’s door. Such an approach may be easier, and thus a faster SAD insertion time may be achieved. The LT was further evaluated by Steinmann et al. [35]. They compared SADs with the Macintosh and Airtraq laryngoscopes. Both of the studied supraglottic airway devices, i.e., the LT and the LMA Supreme, outperformed both laryngoscopes in terms of placement times as well as the ease of use. However, of note is the fact that all devices were assessed by a mixed group of both paramedics and emergency doctors, and this may have an impact on the results. Furthermore, access to the manikin was also allowed through the door with all the above-mentioned advantages.

Data on the performance of the Baska mask in trauma patients are rather limited in the literature. Despite this fact, it was evaluated in military settings [36]. In this study, the Baska mask was compared with tracheal intubation. The authors found that its use was associated with an increased first-pass success as well as higher overall success rates. Furthermore, the Baska mask could be a valuable alternative to standard tracheal intubation. These results were in opposition to the findings of our study, where the Baska device was second to last in regard to the mean T_iv_ of 16.5 s (3.1) vs. 11.2 s (2.4) for the AuraGain. Furthermore, the Baska mask had a low first-attempt success rate of only 78%.

The impact of SADs on the environment is a complex and not fully studied topic. In general, there are three stages at which the environmental impact is seen: 1. the manufacturing process of SADs, 2. the transport of devices to their final destination, e.g., hospital, ambulance station, etc., and 3. their use. The latter can be further subdivided into the reprocessing (for reusable SADs) and waste management of the single-use devices [37]. The data in the current literature regarding supraglottic airway devices is sparse. In a recent study, Talbot et al. evaluated three SADs, both reusable and single-use [38]. They found out that the single-use devices had a higher climate impact than reusable SADs. No studies exist in the current literature regarding the environmental impact of the SADs evaluated in our study. However, all of them were single-use, and therefore, we can assume that they had a higher impact on the environment.

This study has several limitations. First of all, it was carried out using an airway manikin. It has to be emphasised that it may be challenging to extrapolate the results or performance of studied SADs into the human population due to a variety of reasons. Inserting a supraglottic airway device in real patients may be more difficult when compared to an airway manikin. Furthermore, it is difficult to replicate the presence of various secretions, such as saliva or blood, in the manikin model. All of these factors may complicate the use of supraglottic airway devices in humans. On the other hand, one has to be aware that manikin studies are an early but also important stage in the evaluation process of new airway devices [39]. Another limitation is the fairly small number of participants, which consisted of fifty paramedics. Furthermore, a potential source of selection bias exists as our study included fully qualified and experienced paramedics.

## 5. Conclusions

This study has found that the AuraGain was superior in terms of both the insertion to ventilation time as well as the first-attempt success rate. The Baska mask was found to be easier to use. However, the overall performance of the studied devices identified the AuraGain as the supraglottic airway device of choice for airway management in an entrapped trauma patient with restricted access.

## Figures and Tables

**Table 1:** Insertion and ventilation time (T_iv_).

Studied Device	Time Required to Insert the Device and Ventilate the Manikin (T_iv_) [s]			
	Min	Max	Median (IQR)	*p* Value
Baska	10.1	21.8	9 (10)	<0.001
iLTS-D	7.2	30.1	9.5 (11)	0.23
AuraGain	6.7	20.3	7.5 (4)	<0.001
DMSDLLM	9.7	28.9	9 (7)	0.37

IQR—Interquartile Range, s—seconds, iLTS-D—Intubating Laryngeal Tube Suction—Disposable, and DMSDLLM—DM Safety Double-Lumen Laryngeal Mask.

**Table 2:** The efficacy of the studied devices.

Studied Device	Number of Attempts Required for Successful Placement [*n* (%)]		
	1st Attempt	2nd Attempt	3rd Attempt
Baska	39 (78)	9 (18)	2 (4)
iLTS-D	40 (80)	6 (12)	4 (8)
AuraGain	45 (90)	5 (10)	-
DMSDLLM	41 (82)	9 (18)	-

iLTS-D—Intubating Laryngeal Tube Suction—Disposable, and DMSDLLM—DM Safety Double-Lumen Laryngeal Mask.

**Table 3:** User-friendliness of the studied devices.

Studied Device	NRS			
	Min	Max	Mean (SD)	*p* Value
Baska	7	10	8.6 (0.7)	0.195
iLTS-D	5	8	6.8 (1.41)	<0.0001
AuraGain	7	10	8.4 (0.82)	<0.0001
DMSDLLM	6	9	7.7 (0.91)	0.0005

NRS—Numerical Rating Scale, SD—Standard Deviation, iLTS-D—Intubating Laryngeal Tube Suction-Disposable, and DMSDLLM—DM Safety Double-Lumen Laryngeal Mask.

**Table 4:** A summary of the studied devices.

Device	Baska	iLTS-D	AuraGain	DMSDLLM
Type	Supraglottic	Retroglottic	Supraglottic	Supraglottic
Presence of gastric channel	Yes	Yes	Yes	Yes
Is tracheal intubation possible?	Yes	Yes	Yes	Yes
Are paediatric sizes available?	Only ‘large child’	Height > 125 cm	Yes	Yes
Reusability	Single-use	Single-use	Single-use	Single-use
Cuff type	Self-sealing	Inflatable	Inflatable	Inflatable

iLTS-D—Intubating Laryngeal Tube Suction-Disposable and DMSDLLM—DM Safety Double-Lumen Laryngeal Mask.

## Data Availability

Data is contained within the article.
